# Targeting the P2X7 Receptor in Age-Related Macular Degeneration

**DOI:** 10.3390/vision1020011

**Published:** 2017-03-31

**Authors:** Dongli Yang

**Affiliations:** Department of Ophthalmology and Visual Sciences, University of Michigan, 1000 Wall Street, Ann Arbor, MI 48105, USA; dlyang@umich.edu; Tel.: +1-734-763-5322

**Keywords:** P2X7 receptor, age-related macular degeneration (AMD), retinal pigment epithelium, mononuclear phagocyte

## Abstract

The P2X7 receptor (P2X7R) is a membrane receptor for the extracellular adenosine triphosphate (ATP). It functions as a ligand-gated non-selective cation channel and can mediate formation of a large non-selective membrane pore. Activation of the P2X7R induces multiple downstream events, including oxidative stress, inflammatory responses and cell death. Although the P2X7R has been identified in the retinal pigment epithelium (RPE) and different layers of retina, its biological and pathological functions as well as its downstream signaling pathways in the RPE and retina are not yet fully understood. Better understanding of the function of P2X7R in the RPE and retina under normal and disease states might lead to novel therapeutic targets in retinal diseases, including age-related macular degeneration (AMD). This brief review will mainly focus on recent findings on in vitro and in vivo evidence for the role of the P2X7R in the RPE and AMD.

## 1. Introduction

Age-related macular degeneration (AMD) is the leading cause of blindness in the elderly in developed countries, affecting tens of millions of people worldwide. It has two forms: dry AMD (geographic atrophy) and wet AMD (neovascular AMD). The dry AMD is the most common form (80%–90%), while wet AMD is less common (10%–20%) and more severe. In dry AMD, small white or yellowish deposits, termed drusen, typically accumulate between the retinal pigment epithelium (RPE) monolayer and Bruch’s membrane, followed by RPE and photoreceptor degeneration [1,2,3]. In wet AMD, excessive amounts of vascular endothelial growth factor (VEGF) were produced by the RPE and other retinal cells. VEGF contributes to growth of abnormal choroidal blood vessels toward the retinal layers, causing the most severe vision loss due to leakage of blood from abnormal new blood vessels [3,4]. Although wet AMD can be managed by anti-VEGF treatment, currently there is neither a cure nor means of prevention for both forms of AMD. Therefore, there is a critical need to identify new targets for the development of novel therapeutics for treatment of AMD. 

The P2X7 receptor (P2X7R), first discovered in immune cells [5] and then cloned from a rat brain cDNA library [6], has now been shown to be widely expressed in non-ocular epithelial cells [7,8,9] and ocular epithelial cells, including the RPE [10,11,12,13]. The P2X7R is an ATP-gated ion channel that belongs to the family of P2 receptors for extracellular nucleotides. P2 receptors consist of two subfamilies, P2X receptors (P2X1-7) and P2Y receptors (P2Y1,2,4,6,11,12,13,14) [14,15,16]. Special attention has been paid to the role of the P2X7R due to its unique long C terminus and pore formation, and its involvement in oxidative stress, inflammatory processes and cell death. The role of the P2X7R in AMD has previously been reviewed by us [3]. This brief review will mainly focus on recent findings on in vitro and in vivo evidence for the role of the P2X7R in the RPE and AMD.

## 2. The Expression of the P2X7 Receptor in the Retina and the RPE

The P2X7R has been localized to different layers of retina and the RPE. The vertebrate retina is a structure of ten-layers from closest to farthest from the vitreous body: (1) inner limiting membrane (ILM); (2) nerve fiber layer (NFL); (3) ganglion cell layer (GCL); (4) inner plexiform layer (IPL); (5) inner nuclear layer (INL); (6) outer plexiform layer (OPL); (7) outer nuclear layer (ONL); (8) external limiting membrane (ELM); (9) photoreceptor layer; and (10) the RPE layer. 

### 2.1. The Expression of the P2X7 Receptor in the Retina

The P2X7R has been detected in the human, monkey and rodent retina and localized to specific cell types and different layers of the retina. In the monkey retina, immunoreactivity for P2X7R was observed in the INL, IPL, and GCL. In the INL, horizontal cells were strongly immunopositive; in the IPL, fine processes were immunopositive; and in the GCL, the majority of ganglion cells were immunopositive [17]. Another study reported that a strong P2X7 staining was seen in cone photoreceptors of monkey retina [18]. However, whether the P2X7R was expressed in microglia of monkey retina remains controversial. No P2X7R immunoreactivity was observed in microglia of the monkey retina in one study [17], while the P2X7R was primarily expressed in microglia/macrophages of the monkey retina in another study [18]. In the human retina, a study reported that the P2X7R protein was localized to the IPL and OPL, and P2X7R mRNA expression was detected in the inner retina and in the GCL [19]. Human retinal Müller cells [20,21] and human RPE cells [11] were reported to express the P2X7R, while no P2X7 immunoreactivity was observed in Müller cells [17] and the RPE [18] of the monkey retina, suggesting that the distribution of P2X7R in monkey retina is different from that of human retina. 

In the rodent retina, the P2XR is widely expressed in both inner and outer retina. Retinal ganglion cells, amacrine cells, horizontal cells, microglia and photoreceptors all express P2X7R or functional P2X7R [22,23,24,25].

### 2.2. The Expression of the P2X7 Receptor in the RPE

Independent research groups have demonstrated that the P2X7R is expressed in the RPE at both mRNA and protein levels (Table 1). The P2X7R protein was first documented in a human RPE cell line, adult retinal pigment epithelium (ARPE)-19 cells [10]. Later, we and others demonstrated the presence of P2X7R mRNA in ARPE-19 [11,12]. Furthermore, we and others found that P2X7R mRNA is expressed in native human RPE cells [11,13]. Primary cultured human RPE also express both P2X7R mRNA [11,13] and protein [11].

In mice, the P2X7R was found to be present on both apical and basolateral membranes of RPE cells in a C57BL6 mouse at 15 months [12]. To determine the distribution of P2X7R on young and old mouse RPE monolayers in vivo, we used indirect immunofluorescence labeling of P2X7R in mouse retina. We demonstrate that P2X7R protein is expressed on apical and basolateral membranes of the RPE monolayer in both young (4 months old) and old (18 months old) mice, with mainly localized on basolateral membranes [26]. Old mice expressed significantly more P2X7R protein [27]. The increased mRNA expression of P2X7R was also observed in mouse RPE/choroid tissues from ABCA4^−/−^ mice, a model of Stargardt’s retinal degeneration, when compared with control wild type mice [12]. Thus, aging and a degenerative disease condition could increase the vulnerability of RPE cells to extracellular ATP through increased expression of P2X7R.

## 3. Interaction between the P2X7 Receptor and Pannexin-1 Channel

Extracellular ATP concentration reflects the balance between rates of ATP release and extracellular metabolism. Such balance can be altered under pathologic conditions such as inflammation, oxidative stress, infections, cell injury and cell death [28,29,30,31]. Thus, it is possible that ATP released during pathologic conditions is capable of acting on the P2X7R in the RPE cells and other retinal cells in an autocrine or a paracrine manner, similar to cells in other tissues [32,33,34].

Released ATP is degraded by nucleoside-triphosphate diphosphohydrolases (NTPdases). Cultured ARPE-19 cells and native bovine RPE express NTPdases and degrade ATP [35,36]. Unfortunately, no direct comparison of activities of NTPdases between native and cultured RPE in the same species has been reported, nor has the role of NTPdases in AMD been reported.

Activation of the P2X7R opens a membrane channel permeable to small cations (Na^+^, Ca^2+^, K^+^) and changes from a small cation channel to a wider pore that allows the passage of molecules (including ATP) up to 900 Da. At least two hypotheses have been proposed to explain the conversion of a small cation channel to a wider pore. One hypothesis is pore dilation hypothesis that states that this is an intrinsic property of the P2X7R, and the second suggests that the pore is an independent pore-forming membrane protein activated by the P2X7R [37]. Pannexin-1 is identified as a pore-forming membrane protein associated with the P2X7R [38].

Pannexin-1 subunits form hexameric plasma membrane channels that are widely expressed in many mammalian tissues. Each subunit of pannexin-1 channels has four putative transmembrane domains [39]. Pannexin-1 channels are activated by diverse mechanisms such as activation of P2X7R upon ATP addition [38], increased intracellular Ca^2+^ [40], high extracellular K^+^ [41], and proteolytic (i.e., caspases 3, 7 or 11) cleavage of the distal C terminus [42,43,44]. Sequential C-tail removal from individual subunits in hexameric pannexin-1 channels regulates cell permeable to both small ions and large molecules (e.g., fluorescent dyes, ATP) [45]. ATP released through pannexin-1 channels could create a locally high ATP concentration sufficient to activate the P2X7R, contributing to diverse pathophysiological processes such as inflammation and cell death [39,46,47].

Despite pannexin-1 has been shown to interact with P2X7R and to activate the inflammasome in macrophages [38] and in neural cells [41], little is known about the role of pannexin-1 and its signaling pathways in the RPE and AMD. As pannexin-1 physically interacts with the P2X7R, is functionally linked to the P2X7R and can be activated by various mechanisms [38,40,41,42,43,44,45], investigating the interaction between the P2X7R and pannexin-1 channel in the RPE and other retinal cells would be critical to understand the role of P2X7R/pannexin-1 pathways in the RPE and AMD.

## 4. Role of the P2X7 Receptor in In Vitro Models of AMD

Activation of P2X7R can result in different modes of cell death such as apoptosis in RPE cells [11] and photoreceptors [48], apoptosis and necrosis in immune cells [5,6,49] or autophagic cell death in muscle cells [50], depending on doses and duration of P2X7R agonist as well as cell types.

Dysfunction and apoptotic cell loss of RPE are key mechanistic elements in AMD progression [1,2,3,51]. Using primary cultured human RPE cells from different donors, we demonstrated that activation of P2X7R induces human RPE apoptosis that is dependent on P2X7R-mediated Ca^2+^ influx [11]. We proposed that abnormal Ca^2+^ homeostasis through the activation of P2X7R could cause the RPE dysfunction and apoptosis that underlie AMD [11]. Based on literature and our own research, we proposed multiple P2X7R-mediated signaling pathways: Ca^2+^-mitochondrial pathway, NLRP3 inflammasome pathway, and/or phagosome-lysosome pathway, which lead to proinflammary cytokine production and secretion, impaired autophagic degradation, and death of RPE and photoreceptors [3]. Interestingly, in primary cultured myoblasts and myotubes but not in macrophages, ATP induced P2X7R-dependent autophagic flux, leading to caspase-3- and caspase-7-independent cell death. Moreover, heat shock protein 90 (HSP90)-dependent large pore formation, but not Ca^2+^ influx and mitogen-activated protein kinase (MAPK)1-MAPK3 activation, triggered P2X7R-evoked autophagy in myoblasts [50]. It would be interesting to test whether the P2X7R-dependent large pores could depend on HSP90 and trigger autophagy in the RPE under normal and AMD conditions.

To mimic AMD in vitro, amyloid β, one of the main components in drusen, was used to treat ARPE-19 cells [52] or the retinal Müller glial cell line (MIO-M1) [53]. Oxysterols are auto-oxidized forms of cholesterol, including 7-ketocholesterol, 7-β hydroxycholesterol, 24-hydroxycholesterol, 25-hydroxycholesterol and 27-hydroxycholesterol. Olivier et al. measured the levels of oxysterols, after treatment of ARPE-19 cells with 25 μM aggregated amyloid β. Compared to control ARPE-19 cells, aggregated amyloid β significantly increased the levels of 25-hydroxycholesterol and 27-hydroxycholesterol, but did not significantly affect the levels of other three forms of oxysterols. The level of cholesterol was also not affected by the treatment [52]. Both 25-hydroxycholesterol and 7-ketocholesterol induced necrosis of ARPE-19 cells; 25-hydroxycholesterol, but not 7-ketocholesterol, also led to a significant increase in chromatin condensation [52]. The P2X7R mediates the toxicity of oxysterols in ARPE-19 cells [52]. 25-Hydroxycholesterol induces P2X7R activation, as assessed by membrane pore formation using YO-PRO-1 uptake assay. This induced pore formation is inhibited by a pannexin-1 inhibitor, probenecid, while 7-ketocholesterol-induced pore formation was not inhibited by probenecid, suggesting the involvement of pannexin-1 in 25-hydroxycholesterol-, but not 7-ketocholesterol-induced pore formation in ARPE-19 cells [52]. The P2X7R also mediates the toxicity of amyloid β in microglia in in vitro and in vivo models of Alzheimer’s disease [54,55]. Thus, the P2X7R could be the driving force behind oxysterols- and amyloid β-related disorders such as AMD and Alzheimer’s disease.

Retinal Müller glial cells are also implicated in AMD. Treatment of the immortalized human Müller cell line MIO-M1 with amyloid β peptide, Wakx et al. demonstrated that amyloid β peptide induced caspase-independent apoptosis via the activation of the P2X7R [53]. Fish oil rich in polyunsaturated fatty acids, docosahexaenoic acid and eicosapentaenoic acid, combined with a P2X7R antagonist brilliant blue G (BBG), inhibited amyloid β peptide-induced P2X7 pore formation and chromatin condensation in the retinal Müller glial cell line MIO-M1 [53]. 

Mononuclear phagocyte infiltration of the outer blood–retina barrier has been observed in retinal diseases such as AMD. Using co-culture systems to mimic AMD conditions, we demonstrated that immunologically activated mononuclear phagocytes induce intracellular Ca^2+^ signaling and subsequent accumulation of reactive oxygen species that promotes human RPE apoptosis [51]. Greater mouse RPE apoptosis was induced when mononuclear phagocytes were immunologically activated and RPE cells were isolated from Sod2^+/−^ mice [56]. Interestingly, the P2X7R has been identified as a key player in activation of mononuclear phagocytes (e.g., microglia or brain macrophages in the central nervous system). Microglial activation is associated with the pathogenesis and progression of age-related diseases [57]. Inhibition of P2X7R and its downstream interleukin-1β (IL-1β) dampened apoptosis of photoreceptors in a monocyte-retina co-culture system [58]. In monocyte/macrophages or P2X7R-transfected epithelial HEK-293 cells, the P2X7R is identified as a novel scavenger receptor in the absence of its ligand, extracellular ATP [18,59]. Although eleven P2X7R single nucleotide polymorphisms, including P2X7R Gly150Arg variant, are not significantly associated with AMD, the Tyr315Cys variant in the P2X4 receptor (P2X4R) gene is 2-fold more frequent in AMD patients than controls. Furthermore, a rare haplotype with two rare genetic variants (P2X4R Tyr315Cys and P2X7R Gly150Arg) is associated with increased susceptibility to AMD [18]. HEK293 cells expressing wild type P2X7R or P2X7R 150Arg variant confer robust phagocytosis of latex beads, whereas HEK293 cells coexpressing the P2X7R 150Arg with P2X4R 315Cys almost completely inhibit phagocytosis of beads. Fresh peripheral human blood monocytes containing this heterozygous P2X7R 150Arg-P2X4R 315Cys haplotype show a reduction in phagocytosis of beads when compared with WT subjects. The work by Gu et al. implicates a functional interaction between P2X7R 150Arg and P2X4R 315Cys variant receptors could impair the clearance of debris, predisposing individuals toward AMD [18]. However, activation of the P2X7R by ATP not only abolishes P2X7R-mediated phagocytosis function [59], but also leads to cell death in RPE cells [11], photoreceptors [48], and immune cells [5]. Thus, tight control of extracellular ATP concentration is important to maintain a healthy environment for retinal cells.

Taken together, investigating a variety of cell types in culture, suggests that activation of the P2X7R on RPE cells, Müller cells and mononuclear phagocytes may modulate the development of AMD.

## 5. Role of the P2X7 Receptor in In Vivo Models of AMD

Based on in vitro studies, we proposed a role for the P2X7R in AMD in 2011 [11]. Subsequently, the role of P2X7R has been shown in both dry and wet AMD animal models [60,61,62]. Kerur et al. [60] demonstrated that P2X7R is a key protein mediating *Alu* RNA-induced NLRP3 inflammasome activation and consequent RPE degeneration in a mouse model of dry AMD induced by *Alu* RNA. The same group also found in mice that nucleoside reverse transcriptase inhibitors (NRTIs), drugs for human immunodeficiency virus, inhibited *Alu* RNA-induced dry AMD via blocking the P2X7R-mediated NLRP3 inflammasome activation [61]. NRTIs aslo improved outcomes in a laser-induced mouse model of choroidal neovascularization or wet AMD, through blocking the activity of the P2X7R [61,62].

Increased ATP levels were detected in the vitreous samples from wet AMD patients with subretinal hemorrhage [48]. In a mouse model of subretinal hemorrhage, a selective P2X7R antagonist BBG prevents photoreceptor cell apoptosis [48], suggesting that activation of P2X7R by endogenous ATP may mediate photoreceptor cell apoptosis in AMD with subretinal hemorrhage. The P2X7R-dependent photoreceptor apoptosis was also demonstrated by using a P2X7R knockout mouse model [63].

Mice with genetic deletion of Cu, Zn superoxide dismutase (Sod1) showed chronic oxidative stress and developed AMD-like features [64,65]. It is well-known that the P2X7R is a key player in oxidative stress [54,66]. Oxidative stress contributes to AMD pathogenesis. We recently showed that oxidative stress induces cultured human RPE cells to release microparticles that carry drusen components such as CD46 [67]. To see if the P2X7R plays a causative role in oxidative stress-induced AMD, we generated P2X7R/Sod1 double-knockout (DKO) mice [65]. We found that concurrent knockout (KO) of P2X7R prevents microparticle accumulation within RPE/choroid tissues, blocks RPE and retina oxidative stress, and protects against AMD-like defects seen in Sod1 KO mice [65]. Targeting the P2X7R could potentially lead to novel therapies for oxidative stress-driven diseases such as AMD [65].

**Figure 1:**
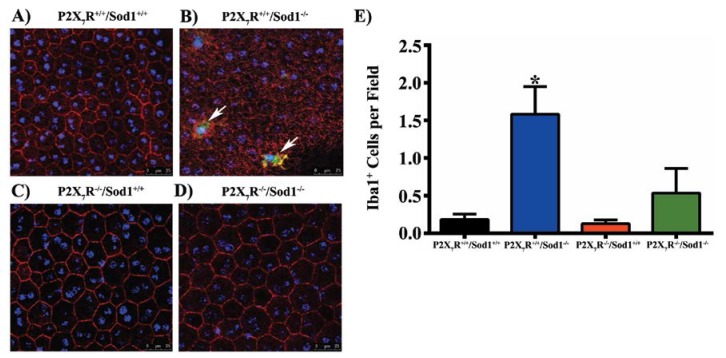
Sod1 knockout causes accumulation of microglia/macrophages and RPE barrier disruption which are prevented by P2X7R/Sod1 double-knockout (DKO). (**A**–**D**) Representative images of RPE flat mounts from mice aged 7 to 12 months, average age 10 months. RPE flat mounts were fixed and stained with rabbit anti-ionized calcium-binding adapter molecule 1 (Iba1), followed by Alexa Fluor 488-conjugated donkey anti-rabbit IgG (Green), Phalloidin-Rhodamine (Red) and Hoechst 33342 (Blue). Twelve random fields were imaged, four from the central, medial, and peripheral regions each, and the number of Iba1^+^ cells (white arrows) were quantified. Scale bars, 25 µm. (**E**) Quantification of Iba1^+^ cells showing the increased number of Iba1^+^ cells in Sod1 KO mice was significantly attenuated in the DKO mice. Data presented as mean ± SEM. *n* = 4–5. * *p* < 0.05 compared to all other groups. From Carver et al. (2017) [65].

Most recently, we found that lack of Sod1 disrupts RPE barrier integrity in vivo, and results in a significant increase in microglia/macrophages in the subretina, while the RPE barrier integrity is maintained and the number of accumulated microglia/macrophages is significantly decreased in mice that lack both P2X7R and Sod1 genes (Figure 1) [65]. Our results indicate that the P2X7R plays a critical role in inducing accumulation of microglia/macrophages in the subretina [65]. Hu et al. reported that mononuclear phagocytes from Cx3cr1-deficient mice increased the P2X7R surface expression, which induces IL-1β maturation and secretion. Inhibition of P2X7R by BBG or its downstream IL-1β by recombinant IL-1Ra which inhibits IL-1 receptor activation, decreased apoptosis of photoreceptors in light-induced subretinal inflammation in Cx3cr1-deficient mice in vivo [58].

In summary, the P2X7R mediates AMD-like defects in several animal models of dry and wet AMD. Genetically knocking out P2X7R or pharmacologically inhibiting P2X7R can improve outcomes in these animal models.

## 6. Conclusions and Future Directions

The recent findings have demonstrated the presence of the P2X7R in the RPE and different layers of retina. The P2X7R in the RPE and retina is functional in vitro and in vivo. This receptor mediates AMD-like defects in in vitro models of AMD and in animal models of both dry and wet AMD.

In addition to acting as either an ion channel or a non-selective large membrane pore, the P2X7R mediates many biological effects such as oxidative stress, inflammation, and three modes of cell death: apoptosis, necrosis and autophagic cell death. Its expression is highly regulated. The function of the P2X7R is also determined by its polymorphisms, splice variants, and interacting proteins. Better understanding of the P2X7R’s functions and regulations in the RPE and retina under normal, aging and AMD conditions may lead to the development of novel preventive and therapeutic strategies for AMD.

## Figures and Tables

**Table 1:** Expression of the P2X7 receptor in the retinal pigment epithelium (RPE).

Cells	mRNA	Protein	Species	Reference
ARPE-19 cell line	n.d. *	+	Human	[10]
	+	n.d.	Human	[11,12]
Primary cultured RPE	+	+	Human	[11,13]
Freshly isolated RPE	+	n.d.	Human	[11,13]
Freshly isolated RPE	+	n.d.	Mouse	[12]
RPE in situ	n.d.	+	Mouse	[12,26]
	n.d.	-	Monkey	[18]

* n.d., not determined; +, expressed; -, not expressed.

## Abbreviations

The following abbreviations are used in this manuscript:

AMD	Age-related macular degeneration
ARPE-19	Adult retinal pigment epithelium-19
ATP	Adenosine triphosphate
BBG	Brilliant blue G
DKO	Double-knockout
D-PBS	Dulbecco’s phosphate-buffered saline
ELM	External limiting membrane
EtBr	Ethidium bromide
GCL	Ganglion cell layer
HSP90	Heat shock protein 90
Iba1	Ionized calcium-binding adapter molecule 1
IL-1β	Interleukin-1β
ILM	Inner limiting membrane
INL	Inner nuclear layer
KO	Knockout
IPL	Inner plexiform layer
MAPK	Mitogen-activated protein kinase
NFL	Nerve fiber layer
NRTIs	Nucleoside reverse transcriptase inhibitors
ONL	Outer nuclear layer
OPL	Outer plexiform layer
P2X7R	P2X7 receptor
RPE	Retinal pigment epithelium
Sod	Superoxide dismutase
VEGF	Vascular endothelial growth factor
